# Cold Plasma Affects Germination and Fungal Community Structure of Buckwheat Seeds

**DOI:** 10.3390/plants10050851

**Published:** 2021-04-23

**Authors:** Jure Mravlje, Marjana Regvar, Pia Starič, Miran Mozetič, Katarina Vogel-Mikuš

**Affiliations:** 1 Biotechnical Faculty, University of Ljubljana, Jamnikarjeva 101, 1000 Ljubljana, Slovenia; marjana.regvar@bf.uni-lj.si (M.R.); katarina.vogelmikus@bf.uni-lj.si (K.V.-M.); 2 Jozef Stefan Institute, Jamova 39, 1000 Ljubljana, Slovenia; pia.staric@ijs.si (P.S.); miran.mozetic@ijs.si (M.M.)

**Keywords:** cold plasma, seeds, grains, plants, common buckwheat, Tartary buckwheat, fungi, decontamination, *Alternaria*, *Didymella*, *Epicoccum*

## Abstract

Crop seeds are frequently colonised by fungi from the field or storage places. Some fungi can cause plant diseases or produce mycotoxins, compromising the use of seeds as seeding material, food or feed. We have investigated the effects of cold plasma (CP) on seed germination and diversity of seed-borne fungi in common and Tartary buckwheat. The seeds were treated with CP for 15, 30, 45, 60, 90, and 120 s in a low-pressure radiofrequency system using oxygen as the feed gas. The fungi from the seed surface and fungal endophytes were isolated using potato dextrose agar plates. After identification by molecular methods, the frequency and diversity of fungal strains were compared between CP treated and chemically surface-sterilised (30% of H_2_O_2_) seeds. CP treatments above 60 s negatively affected the germination of both buckwheat species. A significant reduction in fungal frequency and diversity was observed after 90 s and 120 s in common and Tartary buckwheat, respectively. The filamentous fungi of genera *Alternaria* and *Epicoccum* proved to be the most resistant to CP. The results of our study indicate that CP treatment used in our study may be applicable in postharvest and food production, but not for further seed sowing.

## 1. Introduction

Seeds act as primary inoculum for many fungal diseases [1,2]. Fungi infecting cereals and pseudocereals originate from fields or storage. The first are mainly phytopathogenic fungi, referred to as preharvest fungi, with major genera including *Alternaria*, *Fusarium*, *Cladosporium*, *Rhizopus*, and many others. Storage fungi or postharvest fungi develop during storage, with the genera *Aspergillus* and *Penicillium* being by far the most recurrent. [3]. They can affect seed quality by suppressing germination or causing spoilage of the stored seeds, compromising the whole process of plant production [4].

Buckwheat is a traditional crop grown in Europe and Asia. It originates from Central Asia and is often referred to as an alternative crop. In Slovenia and the larger central-eastern European area, it has been cultivated for centuries. Currently, it is attracting much attention since the interest in functional foods connected to human health concerns is growing rapidly [5,6,7]. Buckwheat is used for flour and groat products [8], and because it is gluten-free, it is safe for consumption by celiac patients [9]. Besides, it has numerous positive effects on human health [6,8], as it has a high nutritional value and is rich in phenolics, of which flavonoids, especially rutin, predominate [10]. Two buckwheat species are usually cultivated throughout the world: common buckwheat (CB; *Fagopyrum esculentum* Moench) and the lesser-known Tartary buckwheat (TB; *Fagopyrum tataricum* Gaertn.). The former is grown in Europe, North and South America, Australia and most of Asia, while the latter is cultivated mainly in the mountainous regions of China, India, Bhutan and Nepal, in harsh climatic conditions. TB was once grown in some regions of Slovenia, but its production waned in the 1980s [6,7]. Nowadays, buckwheat production is rising in Europe and worldwide, mainly due to modest growing demands, which makes it suitable for organic farming [11] in light of the increasing trends towards sustainable and environmentally friendly agriculture with limited use of chemical fertilisers and pesticides.

Contamination with microorganisms, especially fungi, is a critical concern in the cereal industry, not only as a threat to food safety but also because of significant economic losses and irreversible damage, such as reducing the quality, nutritional value and overall appearance of cereals [12,13,14]. The same is true for buckwheat, as fungi are the leading cause of buckwheat diseases at all stages from seed emergence to harvest. Particularly problematic are the soil parasites responsible for seed decay or the rotting of the seedlings [15].

Information on the structure of the fungal communities of buckwheat seeds is scarce. More than 36 different fungal species belonging to more than 22 genera are reported to colonise buckwheat seeds [15,16,17,18]. Many of them produce toxic secondary metabolites—mycotoxins—that are harmful to humans since they can induce acute intoxication leading to severe sickness and even death [19,20]. Chronic exposure to lower doses of these substances can cause genotoxic and mutagenic effects and even some types of cancer, such as colon and oesophageal cancer [21,22,23,24]. It is thus necessary to prevent fungal infections of seeds, not only those intended for sowing but especially those intended for human consumption. Due to the rapid population growth, which is estimated to reach almost 10 billion people by 2050 [25], new approaches are needed to address global food security concerning crops, cereal grains and their products. Thus far, chemical agents such as fungicides and other xenobiotics have been widely used in crop production to eliminate seed-borne pathogens. In this prospect, there is a great need for new, environmentally friendly and economically sustainable technologies to reduce the use of pesticides and other toxic chemicals in plant production. The recently emerging field of cold plasma technology seems to offer a promising solution.

Plasma is an ionised gas consisting of electrons, atoms, ions, radicals and other molecules coexisting with UV photons and visible light. All these components give plasma unique properties, and although it has no net charge, it conducts electricity [26,27]. Energy must be added to the gas, either as thermal or electrical energy (e.g., electric current, electromagnetic radiation), to generate plasma [28]. Cold plasma (CP), also known as “thermodynamic non-equilibrium” plasma, is characterised by a lower electron density and a lower overall temperature compared to thermal plasma [27]. In recent years, the potential of seed treatment by CP has been identified in agriculture, as it can promote seed germination and serve as a means of surface decontamination [29,30,31]. The active chemical species in plasma have efficient antimicrobial properties and can be utilised for surface sterilisation [13,27]. The results of many previous studies suggest that CP could be used as a novel and alternative technology for seed priming and further processing in the food industry [30].

This work reports on the effects of seed CP treatment on the naturally occurring fungal community structure of CB and TB for the first time. Furthermore, the effect of CP exposure time on the germination of both buckwheat species has been investigated to find the optimum conditions for the inactivation of fungal pathogens.

## 2. Results

### 2.1. Effect of CP Treatment on Seed Germination

**Figure 1:**
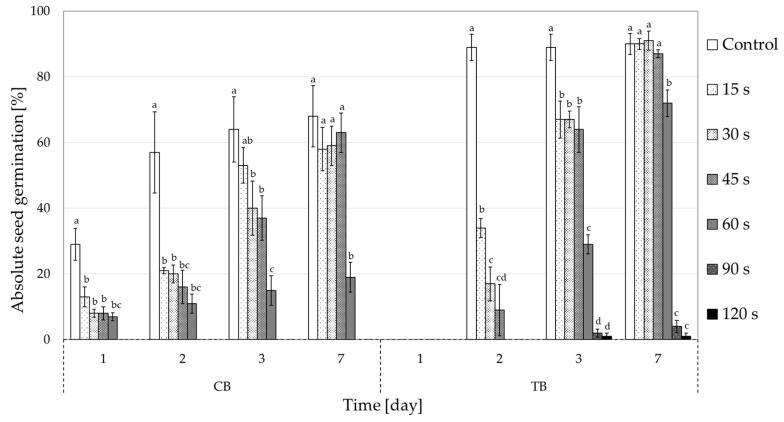
The effects of cold plasma treatments (15–120 s) on seed germination (% of all seed) in both buckwheat species (CB—common buckwheat; TB—Tartary buckwheat) on days 1, 2, 3 and 7. Different letters above the columns represent statistically significant differences between the control group and all cold plasma treatments on a specific day in each buckwheat species. There was no germination in CB after 90 and 120 s CP treatment.

Statistically significant differences in seed germination between all CP treatments (compared to the control group) were observed during the first three days of germination, with the exception of a 15 s CP treatment in CB (Figure 1). This indicates that CP treatment causes a delay in the germination of buckwheat seeds. This can be seen in Table 1, where mean germination time (MGT) and the time to reach 50% germination (T_50_) are calculated. Index T_50_ clearly shows delayed germination with longer CP treatment time in both buckwheat species. This delay was on average at least 1 day in 30 s and longer CP seed treatments. Comparable seed germination percentages were observed after one week (Figure 1) in the control and both buckwheat species treated up to 45 s, while the 60 s treatment caused a significant reduction in germination. Furthermore, the 90 s and 120 s treatments caused a complete suppression of germination in CB and almost complete suppression in TB (4% at 90 s and 1% at 120 s). Interestingly, TB showed a one-day delay in germination compared to CB. In our results only data for untreated seeds (control group) are presented, as the preliminary studies showed no significant difference between the untreated control seeds and the vacuum treated control seeds (data shown in Supplementary Table S1).

**Figure 2:**
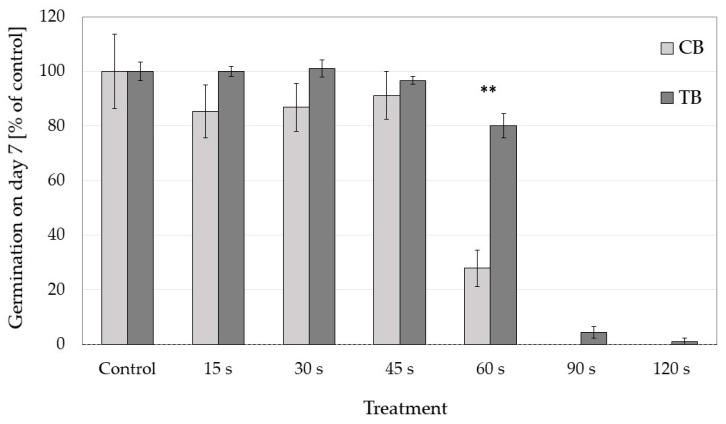
Comparison of the effects of cold plasma treatments (15–120 s) on relative seed germination (% of average control seeds) between both buckwheat species (CB—common buckwheat and TB—Tartary buckwheat) after one week. ** represent a statistically significant difference between buckwheat species (*p* < 0.01).

Relative seed germination (normalised to the average control value) per specific CP treatment showed that both buckwheat species reacted similarly to 15–45 s CP treatments (Figure 2). However, in the 60 s treatment, TB seed germination was on average 50% higher than that of CB. This indicates that TB seeds tolerate longer CP exposures than CB.

### 2.2. Effect of Cold Plasma Treatment on Buckwheat Seed-Borne Fungi

#### 2.2.1. Fungal Colonisation

**Figure 3:**
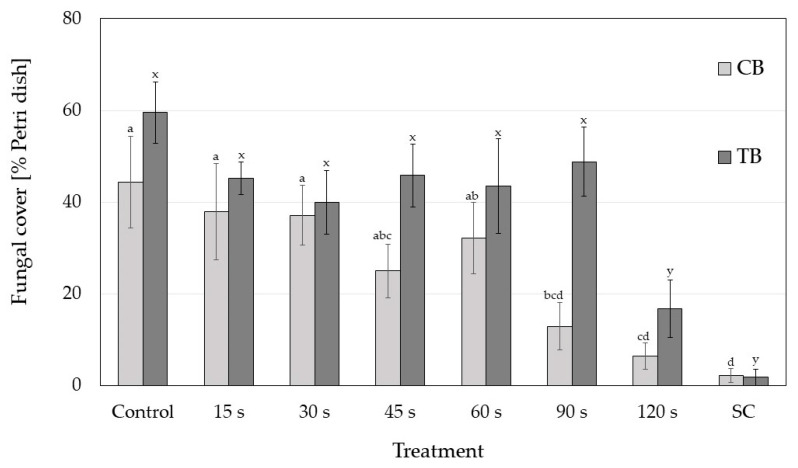
The fungal colonisation level (as mean % of Petri dish area overgrown by fungi) per seed at different cold plasma treatments for each buckwheat species (CB—common buckwheat; TB—Tartary buckwheat). SC—surface-sterilised seeds. Different letters represent statistically significant differences between control and all cold plasma treatments for each buckwheat species (letters a–d for CB and x, y for TB).

In both buckwheat species, CP treatments affected the level of seed colonisation as measured by the percentage of the Petri dish plate overgrown by fungi after one week (Figure 3). The longest CP exposures (120 s) reduced the level of fungal contamination in both buckwheat species (in CB already the 90 s treatment) to the level of the seeds sterilised with 30% of H_2_O_2_ (marked as SC in Figure 3).

**Figure 4:**
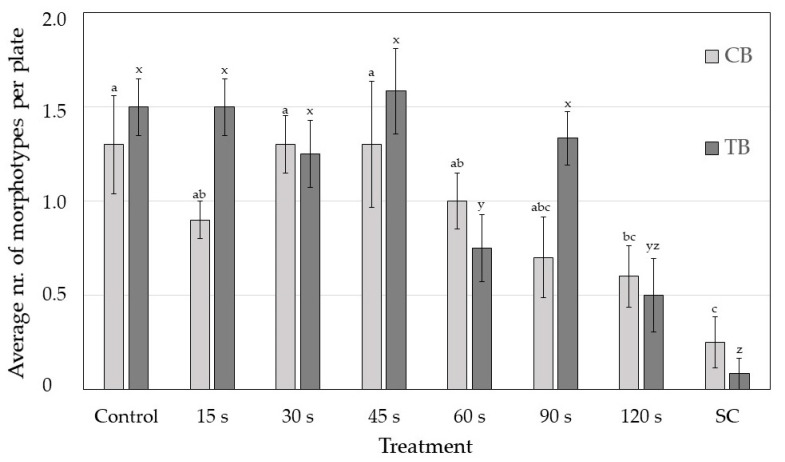
Average number of fungal morphotypes per Petri dish at different cold plasma treatments for each buckwheat species (CB—common buckwheat; TB—Tartary buckwheat). SC—surface-sterilised seeds. Different letters represent statistically significant differences between cold plasma treatments for each buckwheat species (letters a–c for CB and x–z for TB).

The longest exposure of 120 s resulted in a lower average number of fungal morphotypes per plate (Figure 4) in both species. On average, around 1.5 fungal morphotypes were found on each Petri dish in the control seeds of both species and only 0.5 morphotypes after 120 s CP treatment.

**Figure 5:**
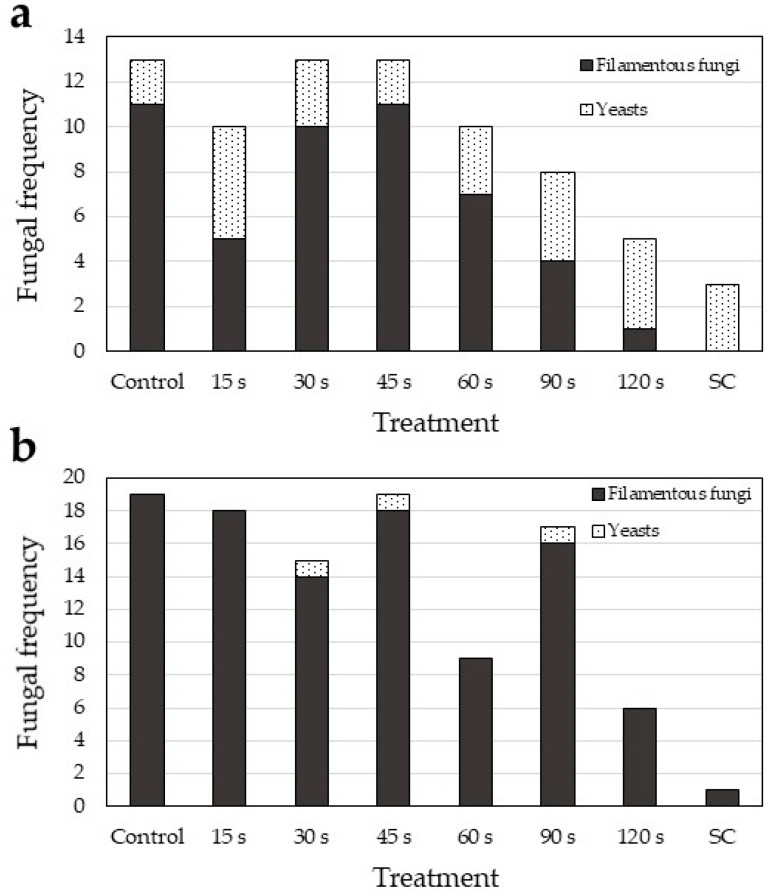
Fungal frequencies per different cold plasma treatments. SC—surface-sterilised seeds. (**a**) Common buckwheat; (**b**) Tartary buckwheat.

The absolute fungal frequencies reveal that lower fungal incidence was found in CB than in TB seeds (Figure 5). In CB, a clear shift was observed from filamentous fungi, which prevailed in shorter CP exposures, to yeasts that were predominant in longer CP exposures. A similar decrease in filamentous fungi was observed in TB seeds. Interestingly, in TB seeds a lower yeast incidence (2.9% of all fungi) was observed than in CB seeds (29.3%).

#### 2.2.2. Fungal Diversity

**Figure 6:**
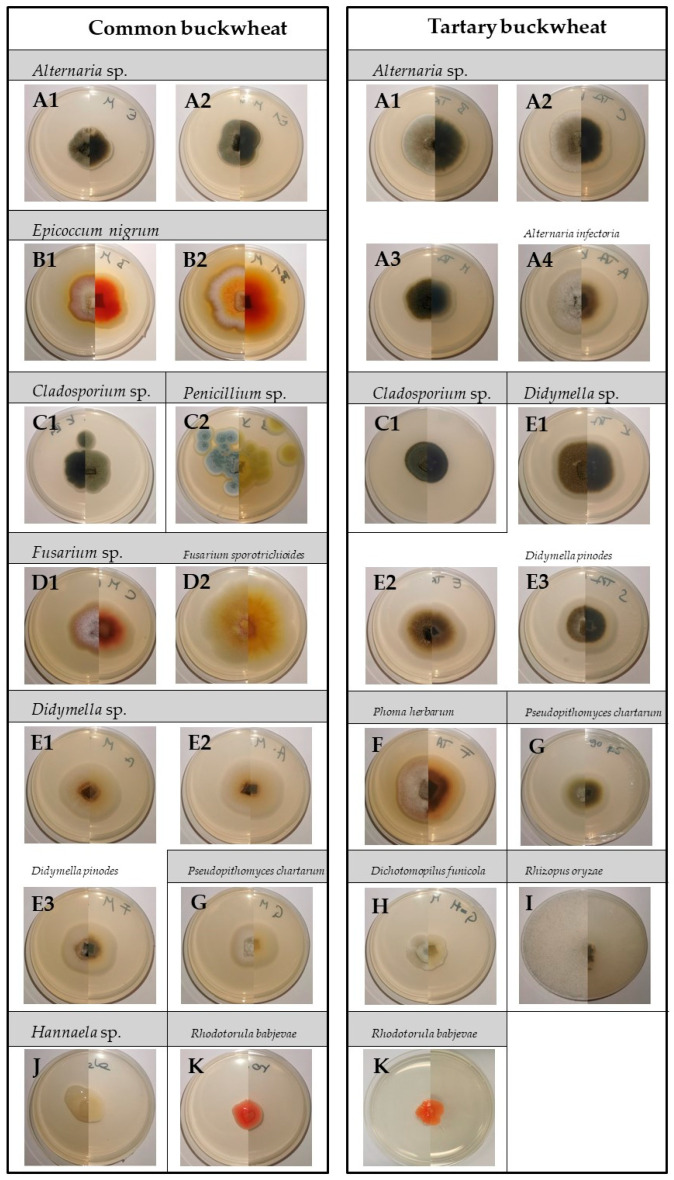
Cultures of fungal morphotypes isolated from common and Tartary buckwheat seeds on potato dextrose agar plates after seven days of cultivation at 22 °C in the dark. The left part of each image shows the front view, and the right part the back view of each morphotype grown in a Petri dish. Different letters represent different morphotypes and numbers indicate different sub-types of each morphotype.

By morphology, the fungal isolates were classified into nine morphotypes and 14 subtypes in CB and seven morphotypes with 13 subtypes in TB (Figure 6). A total of 179 different fungal strains were isolated from buckwheat seeds, of which 75 isolates were obtained from CB and 104 from TB.

Two or three (for more abundantly represented fungi) specimens of each sub-morphotype were selected for molecular identification: a total of 29 from CB and 25 from TB. The sequences were deposited in the GenBank database under accession numbers MW332066—MW332094 (CB isolates) and MW336993—MW337017 (TB isolates) (Supplementary Table S2).

**Figure 7:**
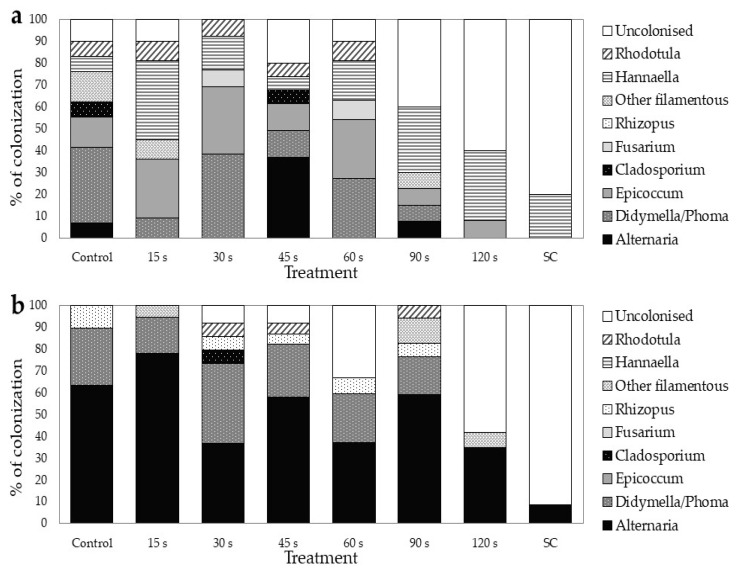
Seed-borne fungal community structure at different cold plasma treatments for (**a**) common buckwheat and (**b**) Tartary buckwheat. SC—surface-sterilised seeds.

The buckwheat seeds harboured a distinct seed fungal community. In the control seeds of TB, genera *Alternaria* and *Didymella* represented the vast majority (90%) of the fungal colonists. In CB, however, there was greater fungal diversity, with the genera *Didymella* and *Epiccocum* comprising more than half of all fungal colonists. The CP treatments affected the seed fungal community structure in both buckwheat species. After 120 s CP treatment, less than 50% of seeds of both buckwheat species were colonised by fungi (Figure 7). Overall, most fungi colonising TB seeds were filamentous fungi, while yeasts accounted for up to 6% of all fungal colonisers (genus *Rhodotorula*), regardless of the CP exposure length. In contrast, yeasts were more common colonisers in CB (predominantly genus *Hannaella*), accounting for between 15% (in control seeds) and 80% (in 120 s CP treatment) of all colonisers. Among filamentous fungi, the genus *Alternaria* predominated in TB seeds, accounting for between 40% and almost 84% of the total fungal community in 120 s CP treatment. We have found at least six morphologically distinct types of *Alternaria* isolates, but only *A. infectoria* was identified down to the species level. In CB, the predominant colonisers belonged to *Didymellaceae*, with *Epicoccum nigrum* accounting for 12%–31% and a species from the genus *Didymella* (formerly known as *Phoma*) accounting for 10%–38% of all fungi. At the same time, *Didymella* species were also the second most common colonisers of TB seeds, accounting for 17% to over 30% of all fungi. As with isolates identified as *Alternaria* sp., *Didymella* species were also morphologically diverse and grouped into six submorphotypes. Interestingly, in TB and CB, the isolates of *Didymella* sp. were found only in up to 90 s CP exposures, which was also true for most other less common fungal genera. This suggests that most of the filamentous fungi contaminating buckwheat seeds may be sensitive to 120 s CP exposure, except for *Alternaria* sp. and *Epicoccum nigrum*. Fungi from the genus *Cladosporium* were also isolated from CB and TB, but less frequently. A common member of the filamentous fungi found in TB was also *Rhizopus oryzae*, which accounted for about 5% to 10% of the total fungal communities per particular CP treatment. Fungi from the genus *Fusarium* were also found in CB seeds, but in smaller amounts (up to 9%). Other species, rarely isolated, included *Phoma herbarum*, *Pithomyces chartarum* and *Dichotomopilus funicola*. In H_2_O_2_ surface-sterilised group of seeds (SC), only *Hannaella* sp. (yeasts) was isolated from CB and *Alternaria* sp. from TB seeds. All molecularly identified taxa of fungi in both buckwheat species are listed in Supplementary Table S1.

## 3. Discussion

### 3.1. Effect of Cold Plasma Treatment on Seed Germination

Significant progress in the use of cold plasma in agriculture has been observed in the last 20 years. Many studies reported possible positive effects of CP treatment on seed and grain production, indicating that CP treatment can stimulate germination, improve seedling vigour and growth dynamics, and also significantly inhibit pathogen growth by reducing surface colonisation [31,32,33,34,35,36,37,38,39]. CP treatment affects different morphological, physiological and biochemical properties of the seeds [40] and can therefore significantly influence the overall plant production. However, most of the studies focused only on the most commonly grown and studied crops worldwide, such as wheat, maize, barley, soybean and chickpea. In chickpea, improved germination has been observed at a shorter CP treatment up to 1 min, while treatments of more than 5 min induced a change in the cotyledon morphology and a consequent loss of seed viability and weaker germination [30]. A positive effect of shorter CP treatments on germination rate and early seedling growth was observed in spring wheat and maize, while no effect was found in blue lupine [37]. Similarly, germination and seedling vigour significantly increased in shorter AP CP treatments (20–50 s) for wheat seeds [38]. Some experiments on AP CP treatment of maize seeds indicate that shorter treatments (40–80 s) have a positive effect on germination [34], while other authors found no significant changes in maize germination at shorter treatments (60–120 s) [39]. On the other hand, it is accepted that longer CP exposure causes a significant decrease in germination and later inhibition of growth parameters in both LP and AP CP systems [33,34,37,38,39]. All these studies confirm that the effectiveness of CP treatment for seed germination varies with the type and condition of the seed and environmental factors such as climate, soil conditions and water availability [31].

To our knowledge, Šera et al. [41] are the only authors who studied the effects of CP treatment in common buckwheat seeds. They found significant effects of different CP plasma devices with air as supply gas and CP exposure lengths on buckwheat germination and early seedling growth. A small positive effect of atmospheric pressure (AP) plasma sustained by gliding arc discharge was observed, and a strong negative effect of Surface Dielectric Barrier Discharge (SDBD) plasma after a shorter (180 s) treatment. With increasing exposure (300 s and 600 s), increasing inhibitory effect on germination and initial growth of buckwheat was confirmed, indicating that buckwheat is very sensitive to CP treatment. In control seeds, the germination rate was 63%, which is similar to our results (68%). In contrast to Šera et al. [41], we found no positive effects of CP treatment on germination in either of the buckwheat species. However, for shorter treatments (15–45 s), the germination rate was comparable to the control group in both species after 7 days. It can thus be concluded that CP treatment of up to 45 s is suitable for buckwheat seeds. On the other hand, Šera et al. observed little positive or no significant effect on germination rate after 180 s of treatment for most of the tested CP devices, with the exception of SDBD [41]. In our experiments, a 60 s CP treatment resulted in significantly reduced germination in both buckwheat species, with CB more susceptible than TB. Longer treatments (90 s and 120 s) completely reduced the germination rate in both species. This is probably due to a higher input power producing a higher electron density, more UV radiation and more heat [42], affecting the buckwheat seeds already at shorter treatments. However, we did not measure temperature during our experiments and this is something that needs to be determined and further studied in future research. In general, TB was more tolerant to longer CP treatments, as 80% of the seeds (relative to the control group) still germinated at 60 s treatment, while the relative germination rate of CB was only about 30%. This could be attributed to higher concentrations of polyphenols in TB [6]. CP treatment acts as a “positive stress” for the plants at shorter exposures, affecting the hormonal activity of the seeds [36] and possibly causing oxidative stress at longer exposures. Differences in flavonoid concentrations and flavonoid metabolism between CB and TB have been previously linked to environmental factors such as increased UV-B radiation in locations where TB was originally cultivated [43]. TB plants originate from higher altitudes and contain higher amounts of compounds that protect against UV radiation, possibly making them more tolerant of UV radiation in CP.

### 3.2. Effect of Cold Plasma Treatment on Fungal Decontamination

The use of gaseous plasma is known as an alternative sterilisation technique [44], and it was suggested as a potential tool for the decontamination of toxic fungi from the seed surface [45]. Plasma sterilisation uses gases that have no germicidal properties and only become biocidal when plasma is formed. Low pressure (LP) plasmas, as in our case, have been considered for biological sterilisation for quite some time [46]. CP treatment generates many antimicrobial active species, including ozone, monoatomic oxygen, free radicals (superoxide, hydroxyl, nitric oxide, etc.) and ultraviolet radiation [45]. Initially, it was proposed that UV photons play a crucial role in microbial sterilisation [47], but a few years later, the contribution of oxygen atoms (as oxidants) was demonstrated [48]. Now it is recognized that microbial destruction results from the synergistic effect of oxygen atoms and UV photons generated by the gas plasma [49]. Microbial sterilisation in CP treatment is, thus, a result of the direct contact of plasma species (positively charged ions and free electrons) and UV radiation, which is still considered the main sterilisation factor of most CP systems.

Some authors have used different types of AP CP devices to reduce native fungi on various types of seeds [30,36,38,39,50]. All conducted studies have found a significant reduction in naturally occurring fungi with increasing duration of CP treatment. However, no study has been yet conducted on the effects of low-pressure CP on native seed-borne fungal microbiota. In our study, a significant reduction of the fungal frequency of seed-borne fungi was observed after 90 s for CB and after 120 s for TB seeds. In both buckwheat species, a fungal reduction greater than 50% compared to the control group was found after 120 s CP treatment. Similarly, a longer treatment time also resulted in a greater fungal reduction in other studies. Some of them were able to completely decontaminate all naturally occurring filamentous fungi and yeasts, as was observed after 120 s treatment in wheat seeds [38] and after 180 s in maize seeds [39] with AP CP. However, we cannot directly compare our results because a different plasma setup system and different seeds were used in our study. Our results also indicate that overall evaluation of fungal contamination of seeds based on Petri dish area covered with fungi is probably not the most accurate, as obvious in the case of CB seeds, where a clear shift between communities of fast-growing filamentous fungi and slowly growing yeast was observed after longer CP exposures.

When considering decontamination by CP, three basic mechanisms synergistically contribute to microbial inactivation in medium and low-pressure CP systems [51]: the direct destruction of genetic material (DNA) by UV radiation; the erosion of microbial matter from the surface of plant cells (atom by atom) by intrinsic photodesorption; and etching caused by reactive species generated in plasma enhanced by UV radiation. In low-pressure plasmas (below 10 mbar), UV photons dominate the microbial inactivation process, resulting in shorter sterilization times. Especially in oxygen-containing plasma mixtures, this effect can be significantly accelerated by the simultaneous erosion of microbial cells by reactive oxygen species. As the erosion of microbial matter progresses, the number of UV photons successfully interacting with microbial DNA increases, resulting in a shorter time required for complete inactivation of the microorganisms [52,53]. This was also confirmed in some studies, all of which indicated that in the first phase, UV radiation plays the primary role, while in the second phase, charged plasma particles become the leading inactivating agent [44,54,55,56].

Our plasma was generated with a radiofrequency (RF) generator in inductively coupled (IC) mode, which is more efficient in microbial decontamination than its alternative, the capacitively coupled mode. This is attributed to higher electron and ion densities formed in IC mode, which ultimately leads to better enhancement of synergistic mechanisms involving electrons (surface etching) [42]. Furthermore, biological materials can be exposed to plasma in two different modes of operation: “direct exposure” (glow), where the treated sample is in direct contact with the plasma and all plasma-generated species are in contact with the sample; and “indirect or remote exposure” (afterglow), in which the sample is placed at a distance from the plasma source or in an adjacent chamber. In the latter, the amount of transmitted heat, as well as some short-lived plasma species reaching the sample, is greatly reduced [46]. In our case, direct exposure was used, leading to greater efficiency of decontamination and resulting in shorter sterilisation times [44,52].

The effectiveness of CP decontamination is, however, also limited by the penetration depth of the UV photons, as they must reach the microbial DNA. Therefore, a shortcoming of CP sterilisation is its dependence on the actual “thickness” of the microorganisms to be inactivated [52] and on the surface structure of the treated material, which can lead to non-homogenous sterilisation [57]. It has been pointed out that the decontamination effect depends not only on the gas used, plasma parameters and exposure length but also on the type of contaminated seeds, where shape and surface play a major role [14,58]. This might be the reason for the better efficacy of CP treatment on CB than on TB in our study. The seeds of TB are more irregularly shaped and have a more wrinkled surface than those of CB, which provides more space for microbial colonisation and makes them less accessible to plasma. Seed wrinkles and crevices have already been mentioned as a possible factor creating a barrier for CP to reach all microflora [59,60]. It was suggested by Mitra et al. [30] to rotate the samples during CP to ensure uniform exposure of the entire seed surface to the plasma. Fungi can also be present in seeds as endophytes, located under the seed coat and cannot be reached by the CP species, strongly influencing the seed sterilization by CP.

Another factor that should be considered when treating thermally sensitive biological samples, such as seeds, is the possible side effect of plasma treatment, namely sample heating. Although it was reported that there was a minimal effect of thermal degradation by LP oxygen CP generated in IC RF mode as in our case [42], some authors pointed out that sample heating could have an influence. Greater sterilisation efficiency with increasing plasma exposure time may depend on oxygen species alone, but sample heating can occur due to radical interactions with the substrate. It was shown that the temperature accelerates the sterilisation and assists the oxygen atoms in the etching process of the bacterial cells. On the other hand, this also limits the exposure time since the surface of the treated object can become strongly heated. Further studies should be conducted to carefully monitor the temperature of biological material during plasma treatment [55].

### 3.3. Effect of Cold Plasma Treatment on Fungal Diversity

In this study, we attempted to determine how CP treatment affects the fungal community structure of buckwheat seeds. Thus far, the information on fungal colonisers of buckwheat seeds is very scarce [15,16,17,18,61]. Our results suggest that CP affects the fungal community structure of the buckwheat seeds, as fewer fungi were isolated and their diversity was reduced with prolonged CP treatment, especially in CB. *Alternaria* sp. and *Didymella* sp. were the predominant fungal colonisers of both buckwheat species. Especially in TB, the genus *Alternaria* was predominant after all CP exposures, with at least four different morphotypes belonging to four different species. However, only *Alternaria infectoria* was clearly distinguished from other *Alternaria* sp. isolates, as it has been discovered that in ITS identification of *Alternaria* species, *A. infectoria* can be clearly distinguished from the *A. alternaria* species complex [62,63]. The latter contains some very similar common plant pathogens indistinguishable in ITS sequencing [64]. For this reason, our isolates were identified as *Alternaria* sp. but are very likely members of *Alternaria* sect. *alternaria*, which includes some of the most common plant saprophytes, such as *A. alternaria*, *A. tenuissima*, and *A. arborescens*. Similarly to our results, the genus *Alternaria* was, on average, the most common coloniser of freshly harvested buckwheat seeds in previous studies [18] together with genera *Botrytis* and *Cladosporium* [17]. *Alternaria* species were also the predominant colonisers of both CB and TB seeds in the study of Kovačec et al., where their proportion increased during storage in comparison to other genera [16]. That can be problematic as *Alternaria* species are known phytopathogenic fungi that cause many plant diseases such as black point of some grain cereals, black rot of tomato, black and grey rot of citrus fruits and many others [21]. *Epicoccum* species were also found on CB seeds [16,17], but not as frequently as in our experiment. Interestingly, we found no *Epicoccum* sp. on TB seeds. This could be a coincidence or the fact that TB seeds were predominantly colonised by *Alternaria* sp., which has been shown to be an antagonistic fungus to *Epicoccum* sp. [16].

Although less frequently isolated, some other genera such as *Cladosporium*, *Penicillium*, *Phoma*, *Pithomyces* and *Rhizopus*, all of which have been previously found in buckwheat seeds [17,18], were also identified during our investigation. *Fusarium* species, the common colonisers of buckwheat seeds [17,18,61] were also confirmed, though, interestingly, only in CP treatments up to 60 s in CB and not found in TB seeds. This is consistent with studies that found *Fusarium* sp. to be one of the most sensitive fungi to AP CP, with a large or almost complete reduction observed after only 60 s [38,39]. That is a good indication since *Fusarium* species are fungal pathogens of many agriculturally important host plants occurring worldwide, known for causing the wilting of young and adult plants of many species such as tomato, onion and garlic [65]. Besides that, they are also capable of producing mycotoxins [65] that are harmful for human health. Not only *Fusarium* sp. but also some other fungi isolated in our experiments, especially the genus *Alternaria*, are capable of producing mycotoxins, with *A. alternata* being one of the most problematic, as it can synthesise many different mycotoxins and intoxicate food and feed [23]. *A. alternata* was also the most frequently isolated fungal pathogen from onion seeds treated with CP, regardless of exposure time [50]. Similarly, it was also the most resistant fungal contaminant of maize seeds to AP CP treatment, with even 300 s treatment time failing to achieve complete devitalisation [39]. In light of this, further studies should be carried out to find the appropriate CP parameters to achieve its complete reduction.

Besides filamentous fungi, yeasts (belonging to the genera *Hannaella* and *Rhodotorula*) were frequently isolated from CB seeds and became predominant during prolonged CP exposure, accounting for more than half of all fungal contaminants after 90 s and 120 s of CP treatment. Interestingly, yeasts were only seldomly isolated from TB seeds. It is known that yeasts and spores are more resistant to CP treatment because they have extremely thick polysaccharide cell walls [45]. Some authors who compared the antimicrobial efficacy of CP treatment also concluded that fungi (especially yeasts) are more resistant to CP treatment than bacteria, as the complete destruction of bacteria was found to occur in shorter periods than fungi [39,57].

CP treatment has many advantages over conventional methods of sterilisation. It minimises damage to the treated materials since they are not subjected to high temperature or pressure (as in steam sterilisation) or radiation and toxic chemicals such as ethylene oxide, thus posing little risk to personnel and the environment [44,45]. The results of many studies conducted so far indicate that CP could be utilised as a new and alternative technology for seed processing, under optimal plasma source and treatment parameters. The optimum exposure time that resulted in a significant reduction in naturally occurring seed-borne fungi without affecting seed viability was found at 10 s CP treatment for the wheat [66] and 20 s CP treatment for sweet basil seeds [67], respectively. In both experiments, an AP CP system was used with air as the feed gas. Within our experimental conditions, we failed to find an optimum CP exposure that would significantly reduce fungal colonisation and maintain a sufficient germination rate of buckwheat seeds. Therefore, this type of CP treatment would be suitable for surface decontamination of buckwheat grains for the food industry but not for agricultural use. As already pointed out, longer CP exposures were successful in reducing naturally occurring fungi on various seeds but were not effective in seed enhancement because of their negative influence on seed germination and viability [36]. This calls for further studies on different CP systems and working parameters to adjust and find the optimum CP treatments for each plant species to be used for commercial purposes.

## 4. Materials and Methods

### 4.1. Buckwheat Seeds Origin

Seeds of CB (*Fagopyrum esculentum* Moench) and TB (*Fagopyrum tataricum* Gaertn.) were obtained from Rangus Mill (located in Šentjernej, south-eastern Slovenia, about 230 m a.s.l.). They were harvested in 2018 and stored under suitable conditions (in a dry and dark environment at room temperature) until the experiments were performed in 2020.

### 4.2. Cold Plasma Treatment

**Figure 8:**
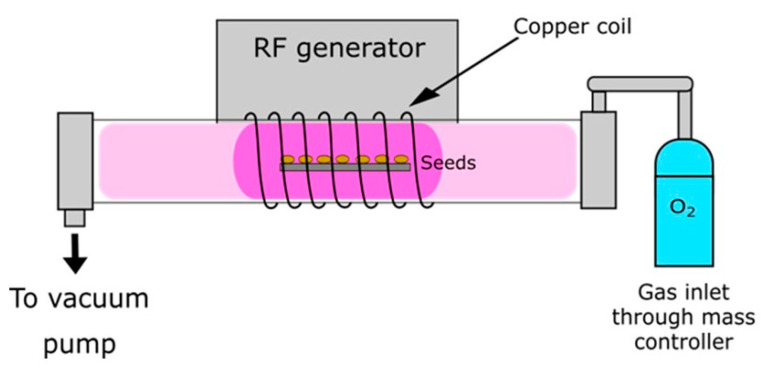
Scheme of the low-pressure cold plasma system using O_2_ as input gas.

**Figure 9:**
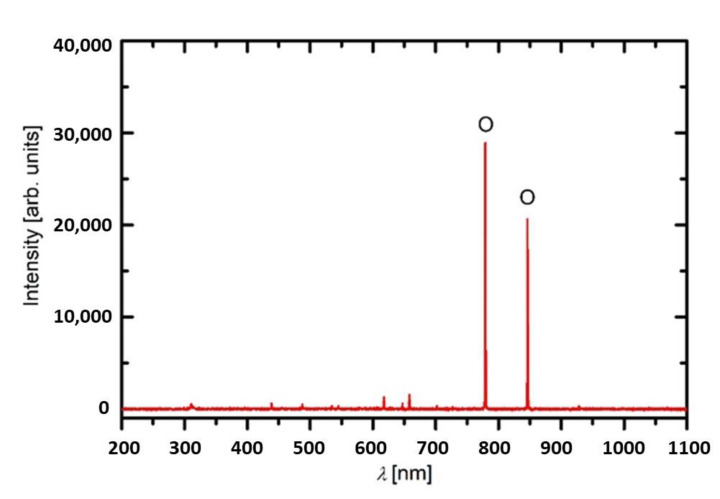
Optical spectrum of oxygen plasma at 50 Pa and 1500 W.

A custom-made large-scale radiofrequency (RF) plasma system [68] was used in our experiments (Figure 8). The system is pumped with a two-stage rotary vacuum pump of pumping speed 80 m^3^·h^−1^. The ultimate pressure achievable in the reactor after the evacuation was about 1 Pa. The system was hermetically tight, so the residual atmosphere contained practically only water vapour. The length of the discharge tube was about 2 m, and the inner diameter 0.18 m, so the volume of the plasma reactor was about 50 litres. A typical optical spectrum is shown in Figure 9. By far the most extensive radiation arises from O-atoms, and there is also a weak molecular band at about 309 nm, which arises from OH-radicals. Other spectral features belong to H-atom transitions (Balmer series). Such a spectrum is typical for highly dissociated oxygen plasma. Seeds, approx. 200 in number, were evenly spread on a metallic mesh and placed inside the tube in the coil area. Plasma was sustained at a pressure of 50 Pa–52 Pa and a power of 1500 W. A copper coil was connected to an RF generator via a matching network. The RF generator operated at a frequency of 27.12 MHz. The power density was, therefore, only 30 W L^−1^. Oxygen gas with a purity of 99.99% was introduced into the plasma system at a constant flow rate of 202 standard cm^3^ min^−1^. The seeds were treated with CP for 15, 30, 45, 60, 90, and 120 s. Immediately after plasma treatment, the seeds were packaged in sterile PVC bags to prevent environmental contamination.

### 4.3. Germination Test

In germination tests, 20 seeds from a given plasma treatment or untreated seeds were placed into Petri dishes (diameter 70 mm) with two layers of filter paper and moistened with sterile distilled water. Each test was performed in five replicates. The seeds were incubated in plant growth chambers at 22 °C, 60% humidity, in the dark. If necessary, sterile distilled water was added to the Petri dishes to maintain constant humidity during germination. The germination rate (GR%) was measured every 24 h for the first three days and then after one week (on the 7th day). It was calculated according to the following Equation (1). The penetration of the radicle through the seed coat was used as the criterion for germination.

(1) GR (%)=(number of germinated seeds)/(total number of seeds) × 100

Mean germination time (MGT) was calculated according to the equation of Ellis and Roberts (1981) [69] as under:

MGT = ΣD × n/Σn(2)

where, n is the number of seeds, which were germinated on day D, and D is the number of days counted from the beginning of germination.

The time to 50% germination (T_50_) was calculated according to the following formula of Coolbear et al. (1984) modified by Farooq et al. (2005) [70] as:

T_50_ = t_i_ + [(N/2 − n_i_) (t_i_ − t_j_)]/(n_i_ − n_j_) (3)

where N is the final number of germination and n_i_, n_j_ cumulative number of seeds germinated by adjacent counts at times t_i_ and t_j_ when n_i_ < N/2 < n_j_.

### 4.4. Effect of CP on Seed-Borne Fungi Decontamination

To test the effect of CP treatment on buckwheat seed-borne fungi, seeds from all CP treatments and untreated seeds were analysed as described below. Chemically surface-sterilised seeds (with 30% H_2_O_2_ and 2 drops of Tween 80 for 20 min) were analysed in parallel to assess the efficacy of the CP treatment as a surface decontaminant.

### 4.5. Cultivation of Seed-Borne Fungi

The agar plate or “Ulster” method was used to assess seed colonisation and fungal growth [71]. Seeds were planted on sterile plastic Petri dishes (diameter 70 mm) filled with 2% potato dextrose agar (PDA) supplemented with the antibiotic chloramphenicol (50 mg L^−1^) to prevent bacterial growth. One buckwheat seed was placed in the centre of each Petri dish to assess fungal growth. Plates were incubated in growth chambers at a temperature of 22 °C in dark conditions for one week.

### 4.6. Seed Colonisation and Morphological Characterisation of Fungi

After one week of cultivation, the plates were examined to measure the percentage of colonised seeds, average fungal area and fungal diversity (morphotypes). Fungal area per plate was determined at day 7 from the mycelial radius and expressed as the percentage of the colonised area diameter relative to the diameter of the Petri dish. Fungi were visually examined and grouped by their morphological appearance (morphotypes and sub-morphotypes). The percentage of colonised seeds (CS), the average fungal area (FA), the average number of morphotypes (MN) and the frequency of each morphotype (MF) for each treatment were calculated according to Equations (4), (5), (6) and (7), respectively:

(4) CS (%)=(number of colonised seeds)/(total number of seeds) × 100

(5) FA (%)=(diameter of fungal area [mm])/(diameter of Petri dish [mm]) × 100

(6) MN=(∑number of morphotypes per plate)/(total number of morphotypes)

(7) MF (%)=(∑isolates of the morphotype)/(∑all fungal isolates) × 100

Fungal isolates were repeatedly sub-cultured until pure cultures were obtained.

### 4.7. Molecular Identification

For molecular identification, at least 2 (3 for more frequent fungi) pure culture isolates of each sub-morphotype were selected (if possible, from different CP treatments). Fungal material was scraped from agar plates and crushed in liquid nitrogen using a mortar and pestle. Fungal genomic DNA was isolated using GenElute^®^ Plant Genomic DNA Miniprep Kit (Sigma-Aldrich, St. Louis, MO, USA) according to the manufacturer’s instructions. Fungal DNA (ITS region) was amplified using the primer pair ITS1f/ITS4 and Taq polymerase in an MJ Research^®^ thermal cycler. The reaction mixtures and PCR conditions were performed according to Likar and Regvar [72]. Purification and sequencing of the PCR products were performed by Macrogen Inc. (Amsterdam, The Netherlands). For further identification, the sequences were subjected to a nucleotide BLAST^®^ search within the NCBI database. After successful identification, the sequence data were processed using the MEGA-X software and submitted to the GenBank database.

### 4.8. Statistical Analysis

The reported results are expressed as mean ± standard error (SE) of five replicates (for germination tests) and at least ten replicates (for fungal growth analyses). Statistical significance between groups (treatments) was determined using a one-way analysis of variance (ANOVA) with Duncan’s post hoc test (Statistica StatSoft version 7). The significance level was considered at a *p*-value of less than 0.05.

## 5. Conclusions

Chemical agents, such as fungicides and other xenobiotics, have been widely used in crop production to eliminate seed-borne pathogens. There is a great need for new, environmentally friendly and economically sustainable technologies to reduce the use of pesticides and other toxic chemicals in plant production.

The results presented in this paper show that low-pressure cold plasma treatment affects germination and seed-borne fungal community structure in seeds of common and Tartary buckwheat species. Shorter exposures (up to 45 s) can be safely applied, as they had no significant effect on seed germination in either of the buckwheat species, while longer exposures reduced the germination rate in both species. TB seeds were found to be more resistant to longer CP exposures than CB seeds.

Only the longest CP exposures (120 s) significantly affected the degree of fungal colonisation and diversity in seeds of both buckwheat species. The genera *Alternaria*, *Didymella* and *Epicoccum* were the most common fungi isolated from the seeds. *Alternaria* was prevalent in TB, and our results suggest that the genus is (beside genus *Epicoccum*) also the most resistant to the given CP treatment. This may prove problematic as *Alternaria* species are known sources of mycotoxins harmful to humans and animals.

CP treatment in general seems to be a promising alternative to remove surface contamination and improve seed germination, thus increasing the overall plant production. However, further research is needed to define the optimum CP treatment parameters and adequately address the problem of contaminated seeds to ensure global food security, provide safer food, and reduce the impact of agricultural production on the environment.

## Figures and Tables

**Table 1:** Mean germination time (MGT) and time to reach 50% germination (T_50_) calculated for both buckwheat species per each CP treatment. Different letters indicate statistically significant differences between control and CP treatments per each buckwheat species. *n.d.*—indices could not be calculated due to very low germination (only few seeds per treatment). Different letters (superscripts) represent statistically significant differences between the control group and cold plasma treatments.

	CB		TB	
Treatment	MGT	T_50_	MGT	T_50_
Control	3.77 ^a^	1.30 ^a^	4.01 ^a^	1.51 ^a^
15 s	4.26 ^bc^	1.56 ^a^	4.71 ^b^	2.33 ^ab^
30 s	4.54 ^cd^	2.41 ^b^	4.99 ^b^	2.54 ^b^
45 s	4.82 ^d^	2.88 ^b^	5.17 ^b^	2.62 ^b^
60 s	3.98 ^ab^	2.40 ^b^	5.86 ^c^	3.63 ^c^
90 s	*n.d.*	*n.d.*	5.89 ^c^	3.67 ^c^
120 s	*n.d.*	*n.d.*	*n.d.*	*n.d.*

## Data Availability

The genetic data presented in this study are in a publicly accessible repository available in Supplementary Table S1.

## Supplementary Materials

The following are available online at https://www.mdpi.com/article/10.3390/plants10050851/s1, Table S1: Preliminary germination tests, Table S2: Molecular identifications of isolates.
