# Supplementary material for: Cold Plasma Affects Germination and Fungal Community Structure of Buckwheat Seeds
Source: Plants (Basel). 2021 Apr 23;10(5):851. doi: 10.3390/plants10050851 (PMC8145130; doi:10.3390/plants10050851)
Supplement: Supplementary file 1 [file plants-10-00851-s001.zip › plants-1149128-supplementary.pdf]

Table S1. Preliminary germination results (% of germinated seeds after 1 week) of control seeds and vacuum treated seeds of both common and Tartary buckwheat (expressed as mean ± standard error). Results were not statistically significant in either buckwheat species.

	Common Buckwheat	Tartary Buckwheat
**Control Seeds**	59.0 ± 3.0	91.0 ± 3.4
**Vacuum Treated Seeds**	52.0 ± 4.3	90.0 ± 2.6
**Significant**	*No*	*No*

Table S2. GenBank accession numbers of the fungal sequences isolated from the common buckwheat (CB) and Tartary buckwheat (TB) seeds, their taxonomic identification together with the nearest GenBank match and % of identity. E-value was 0 in all matches respectively.

Morphotype		Isolate	Taxon	Our GenBank	Nearest GenBank Match	
Group	Subgroup			Acc. No.	Acc. No.	% Ident.
A	A1_CB	NA002	*Alternaria sp.*	MW332093	MN944587.1	99
		NA003	*Alternaria sp.*	MW332092	KY951909.1	99
		NA029	*Alternaria sp.*	MW332069	MK432746.1	99
	A1_TB	TA002	*Alternaria sp.*	MW337015	MT446185.1	99
		TA005	*Alternaria sp.*	MW337013	MN836593.1	100
		TA033	*Alternaria sp.*	MW336995	MF475920.1	100
	A2_CB	NA007	*Alternaria sp.*	MW332088	MN836593.1	100
		NA008	*Alternaria sp.*	MW332087	KY951909.1	99
	A2_TB	TA004	*Alternaria sp.*	MW337014	MT446076.1	100
		TA007	*Alternaria sp.*	MW337012	MT446184.1	100
		TA017	*Alternaria sp.*	MW337004	MF475920.1	99
	A3_TB	TA016	*Alternaria sp.*	MW337005	MK732102.1	99
		TA025	*Alternaria sp.*	MW336999	MG250470.1	100
	A4_TB	TA001	*Alternaria infectoria*	MW337017	MH205934.1	100
		TA001a	*Alternaria infectoria*	MW337016	MK461063.1	99
B	B1_CB	NA004	*Epicoccum nigrum*	MW332091	KT223320.1	99
		NA005	*Epicoccum nigrum*	MW332089	KX869952.1	98
		NA028	*Epicoccum nigrum*	MW332070	MG602540.1	99
	B2_CB	NA020	*Epicoccum nigrum*	MW332077	KX869952.1	99
		NA022	*Epicoccum nigrum*	MW332076	KT898754.1	99
		NA023	*Epicoccum nigrum*	MW332075	KT898567.1	100
C	C1_CB	NA006	*Cladosporium sp.*	MW332089	MN275867.1	97
		NA036	*Cladosporium sp.*	MW332066	MF476044.1	99
	C1_TB	TA029	*Cladosporium sp.*	MW336997	FR799495.1	99
	C2_CB	NA011	*Penicillium sp.*	MW332085	KJ527027.1	98
D	D1_CB	NA030	*Fusarium sp.*	MW332068	MT530119.1	99
	D2_CB	NA001	*Fusarium sporotrichioides*	MW332094	MK562070.1	97
E	E1_CB	NA014	*Didymella sp.*	MW332082	KX017275.1	99
		NA015	*Didymella sp.*	MW332081	MH257396.1	99
		NA018	*Didymella sp.*	MW332079	KX017275.1	99
	E1_TB	TA012	*Didymella sp.*	MW337009	MH257382.1	99
		TA020	*Didymella sp.*	MW337002	MH257382.1	98
		TA023	*Didymella sp.*	MW337000	KF493958.1	100
	E2_CB	NA012	*Didymella sp.*	MW332084	KX017275.1	99
		NA016	*Didymella sp.*	MW332080	KX017275.1	99
	E2_TB	TA013	*Didymella sp.*	MW337008	MH257396.1	99
		TA021	*Didymella sp.*	MW337001	MH257396.1	99
		TA027	*Didymella sp.*	MW336998	AY183371.1	99
	E3_CB	NA013	*Didymella pinodes*	MW332083	FJ194523.1	98
		NA035	*Didymella sp.*	MW332067	KT223339.1	99
	E3_TB	TA018	*Didymella sp.*	MW337003	KX017275.1	99
		TA030	*Didymella pinodes*	MW336996	FJ194522.1	99
F	F_TB	TA015	*Phoma herbarum*	MW337006	KP739881.1	99
		TA014	*Phoma sp.*	MW337007	KX017275.1	100
G	G_CB	NA019	*Pseudopithomyces chartarum*	MW332078	MF476065.1	100
	G_TB	TA034	*Pseudopithomyces chartarum*	MW336994	KM979510.1	99
H	H_CB	NA010	*Dichotomopilus funicola*	MW332086	MH857547.1	99
I	I_TB	TA009	*Rhizopus oryzae*	MW337011	MN006420.1	99
		TA010	*Rhizopus oryzae*	MW337010	MN006420.1	99
J	J_CB	NA024	*Hannaella sp.*	MW332074	KY103515.1	99
		NA026	*Hannaella sp.*	MW332072	HG937070.1	99

		NA027	*Hannaella sp.*	MW332071	KY103515.1	98
K	K_CB	NA025	*Rhodotorula babjevae*	MW332073	KY104719.1	95
	K_TB	TA037	*Rhodotorula babjevae*	MW336993	KY104719.1	95
